# Inflammatory Myofibroblastic Tumor of the Larynx:A Case Report

**Published:** 2016-01

**Authors:** Farzad Izadi, Hadi Ghanbari, Mohammad Reza Azizi, Shahram Gasembaglou, Mohammad Javad Manteghi, Azadeh Ghanbari

**Affiliations:** 1 *Department of Otorhinolaryngology, Hazart Rasoul Akram Hospital, Iran University of Medical Sciences, Tehran, Iran. *; 2 *ENT & HNS Research Center, Hazart Rasoul Akram Hospital, Iran University of Medical Sciences, Tehran, Iran. *; 3 *Pathologist, Shahriyar Hospital, Tehran, Iran. *; 4 *General Practitioner, Iran University of Medical Sciences, Tehran, Iran. *; 5 *College of Dentistry, Ajman University of Science and Technology, Ajman, UAE. *

**Keywords:** Head and neck tumor, Immunohistochemstry (IHC), Inflammatory myofibroblastic tumor, larynx, laryngeal pseudotumor, Laryngeal mass.

## Abstract

**Introduction:**

Inflammatory myofibroblastic pseudotumors are initially described in the lung and various extrapulmonary sites such as the orbits, palatine tonsils, ears, gingiva, pterygomaxillary space, and periodontal tissues. These tumors rarely involve the larynx and predilection to the glottis occurs in an indolent manner.

**Case Report:**

This case describes a laryngeal myofibroblastic tumor in a 46-year-old woman who presented with an aggressive tumor that extended to the floor of the mouth and the base of the tongue. Extended supraglottic laryngectomy was undertaken for the patient. The diagnosis was spindle cell proliferation with dense lymphoplasma cell infiltration compatible with inflammatory myofibroblastic tumor (Inflammatory pseudotumor or plasma cell granuloma). Definitive diagnosis was achieved with immunohistochemical (IHC) staining.

**Conclusion:**

We believe that further IHC studies are required to define the true nature of these tumors especially for those that behave in an aggressive pattern.

## Introduction

Inflammatory myofibroblastic tumors (IMFT) represent a heterogeneous group of unusual pseudo neoplastic lesions that appear to encompass a wide spectrum of histopathological features ranging from plasma cell rich to a high presence of myofibroblasts (1,2). Although IMFT was initially described in the lung (3), it has subsequently been reported in various extrapulmonary sites including the head and neck. IMFT is a benign fibroinflammatory mass of unknown etiology, and the true nature of IMFT is only now beginning to be elucidated (4-6). IMFT has many synonyms: plasma cell granuloma, inflammatory pseudotumor, xanthogranuloma, histiocytoma, and myofibrohistiocytic proliferation. IMFT was first described in 1992 and presents many diagnostic challenges especially in the differentiation from a benign reactive process to a malignant one; however the diagnostic process could be further elucidated by observation of the behavior of IMFT and by further pathological findings. 

## Case Report

A 46-year-old woman presented with symptoms of potato voice and globus sensation for 4 years with no history of trauma or previous operation. She underwent a direct laryngoscopy, which revealed a thickness of the aryepiglottic fold and vallecula on the right side with a submucosal mass at the laryngeal surface of the epiglottis. The patient underwent a conservative laser resection of the involved area. Pathology showed fibrosis only, without any presence of atypias. One and half year later, symptoms recurred. Another laser procedure was undergone but the report remained the same. In the last 2 years, tumor growth occurred so rapidly that a CO2 laser transoral resection was preformed twice.

**Fig 1:**
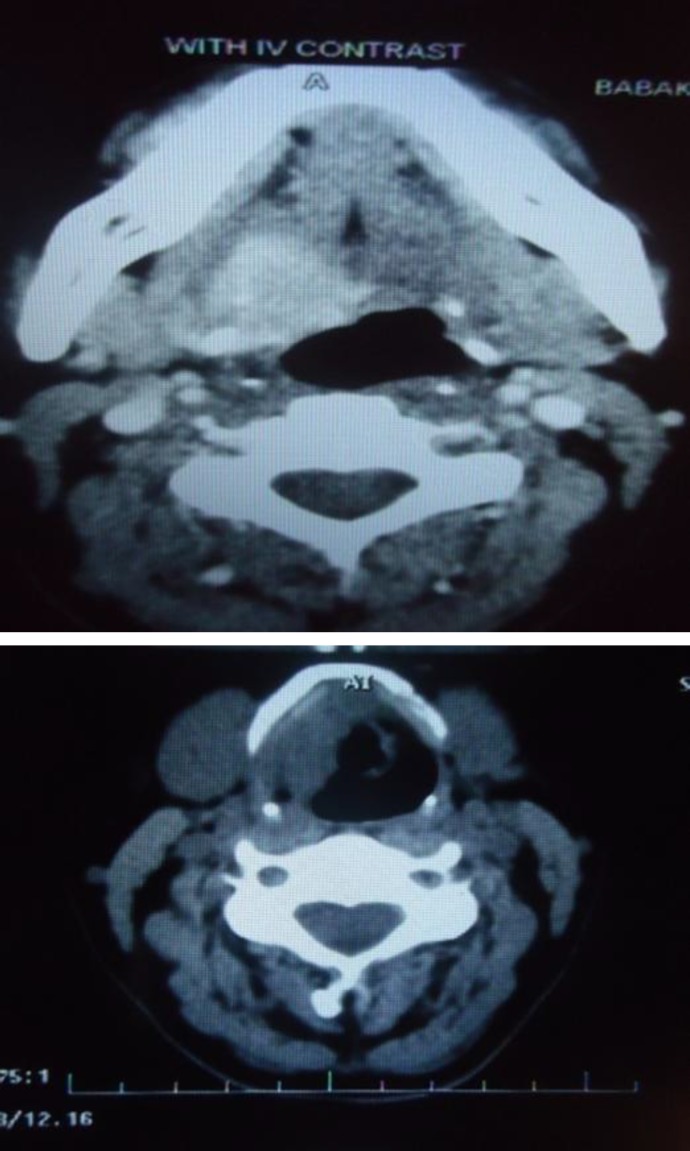
right uptake at the base of tongue (Upper) involvement of the superior part of the layngx((lower).

Unfortunately, endoscopic photos of the preoperative intra laryngeal surgeries do not exist. Before the open surgery, pathology showed squamous and respiratory type mucosa with superficial edema and fibrosis of the deep stroma with no mucosal epithelium atypia. CT showed a large heterogeneous enhanced mass, 30×24 mm in size, located on the right side of the base of the tongue with involvement of the genioglossus, hyoglossus, and mylohyoid muscles. There was a posterior-inferior extension of the mass to the vallecula and pre-epiglottic space; in addition to a thickened aryepiglottic fold in the presence of intact fat planes (Fig.1). 

**Fig 2:**
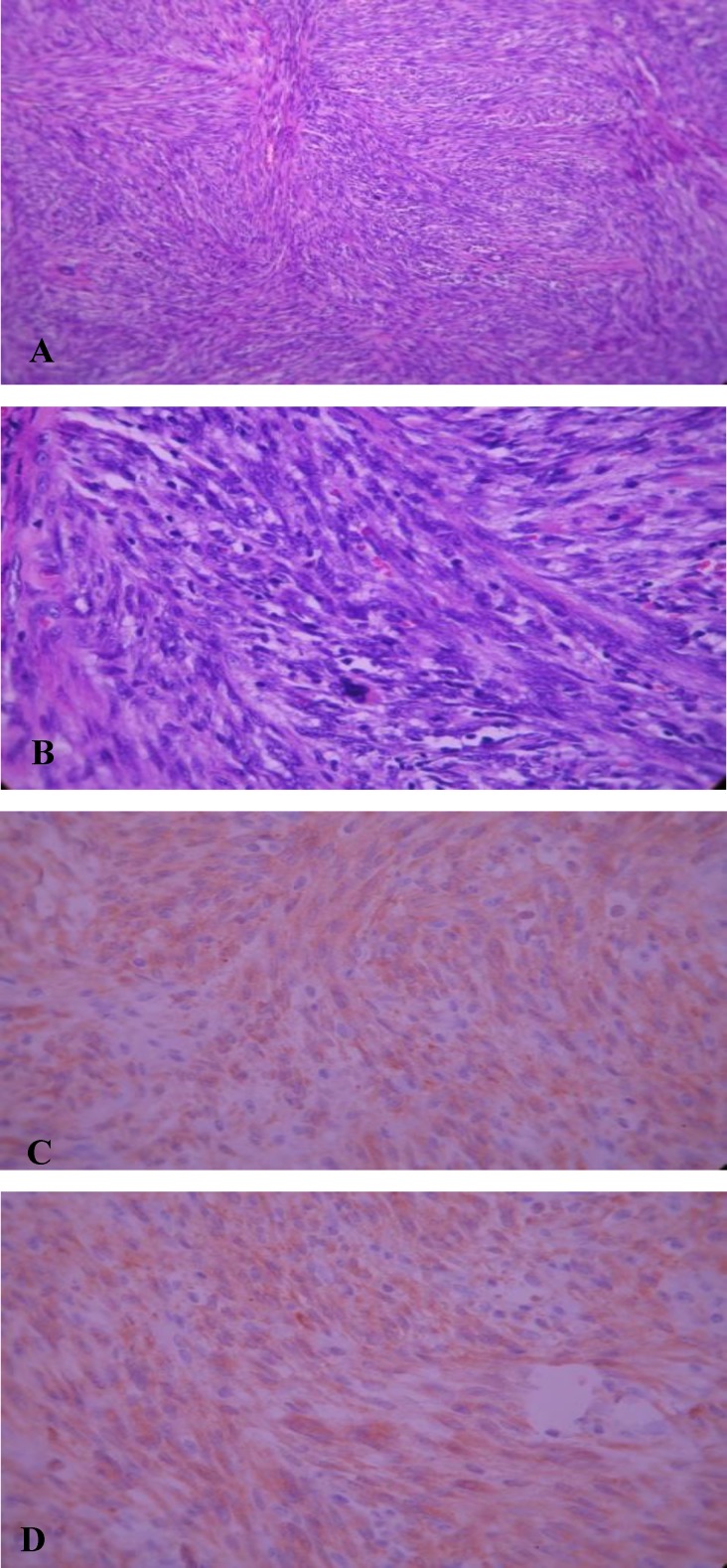
Microscopic: sections show dense spindle cell proliferation with storiform growth pattern associated with patchy infiltration of lymphoplasma cells (a,b), and positive reaction of tumor cells with SMA(c) and Alk-1(d

Upon admission, the patient had a stridor at rest due to the involvement of the base of the tongue and supraglottic region. The patient underwent extended supraglottic laryngectomy due to repeated recurrence and surgeries. The surgical area was primarily reconstructed because of the unilateral mass. This mass presented as an irregular-shaped piece of rubbery, which was a firm and grayish brown tissue, measuring 3×2.5×2 cm and was sent for evaluation. Immunohistochemical staining showed spindle cell positive reaction for SMA and ALK. No reaction for CD34, AE1/3 or desmin was observed; but BcL2 showed a positive reaction in inflammatory cells (Dr Nassizade, Iranian Blood Transfusion organi zation,IHC Lab. Ref#90-590)(Fig.2 a,b,c, d).

The diagnosis was spindle cell proliferation with dense lympho- plasma cell infiltration compatible with inflammatory myofibro- blastic tumor (Inflammatory pseudotumor or plasma cell granuloma). 

## Discussion

IMFT, termed by Weing et al., indicates a proliferative myofibroblastic growth. IMFT has been reported throughout the body under a variety of names and the majority of the lesions follow a benign clinical course and rarely reoccur locally. IMFT should be considered when histological findings from an inflammatory mass are inconclusive. Development of this pseudotumor in the head and neck should always be kept in mind otherwise the diagnosis could be difficult. The etiology and pathogenesis of IMFT is still a mystery. Some investigators consider it to be an immunological host response to many different stimuli; but cytogenetic and molecular studies have pointed to the possibility that at least some subsets of IMFT are, in fact, true neoplasms. However, one subset of IMFT is mostly probably associated with infection. 

Histologically, IMFTs are composed of myofibroblastic spindle cells that are mixed with a prominent infiltrate of lymphocytes, plasma cells, and acute inflammatory cells. The three basic histological patterns were described by Coffin et al and are as follows: myxoid, vascular, and inflammatory areas resembling nodular fascitis; compact spindle cells with intermingled inflammatory cells resembling fibrous histiocytoma; and dense plate-like collagen resembling a desmoid or fibrous scar (7,8). The main differential diagnoses are epidermoid carcinomas with spindle-shaped cells, malignant mesenchy- matous tumors (fibrosarcomas, chondros- arcomas, histiocytomas, and others), and lymphomas. True IMFTs must be distingui- shed from inflammatory pseudotumors developing in response to a healing process, injury, or infection. Treatment options include laser assisted endoscopic excision, high dose steroids but rarely radiation and open excisions. Endoscopic excision with or without steroids is considered to be the first line of treatment as only few recurrences have been reported (9). In this case, the supraglottic inflammatory myofibroblastic tumor had an invasive behavior and progressive nature with extension to the base of the tongue and floor of the mouth (Fig.1). After several conservative CO2 laser surgeries, the patient had to undergo open surgery(extended supraglottic laryngectomy) due to the involvement deep into the supraglottic region and the presence of progressive symptoms. However, the patient was discharged with no morbidity, even though the resection involved the unilateral base of the tongue. Up to now, the patient has been symptom free. 

## Conclusion

IMFTs of the larynx are uncommon and carry a good prognosis but further IHC studies are required to define the true nature of these tumors and to categorize the aggressive subtypes of this tumor.
